# Functional Analyses of Four Cryptochromes From Aquatic Organisms After Heterologous Expression in *Drosophila melanogaster* Circadian Clock Cells

**DOI:** 10.1177/07487304241228617

**Published:** 2024-03-28

**Authors:** Chenghao Chen, T. Katherine Tamai, Min Xu, Libero Petrone, Paola Oliveri, David Whitmore, Ralf Stanewsky

**Affiliations:** * Department of Cell and Developmental Biology, University College London, London, UK; † Department of Neurobiology, University of Massachusetts Chan Medical School, Worcester, Massachusetts, USA; ‡ Department of Psychiatry and Biobehavioral Sciences, David Geffen School of Medicine, University of California, Los Angeles, Los Angeles, California, USA; § Department of Genetics, Evolution and Environment, University College London, London, UK; || Australian Institute of Tropical Health & Medicine, James Cook University, Townsville, QLD, Australia; ¶ Institute for Neuro- and Behavioral Biology, University of Münster, Münster, Germany

**Keywords:** circadian clock, Danio rerio, *Strongylocentrotus purpuratus*, timeless, luciferase, period

## Abstract

Cryptochromes (Crys) represent a multi-facetted class of proteins closely associated with circadian clocks. They have been shown to function as photoreceptors but also to fulfill light-independent roles as transcriptional repressors within the negative feedback loop of the circadian clock. In addition, there is evidence for Crys being involved in light-dependent magneto-sensing, and regulation of neuronal activity in insects, adding to the functional diversity of this cryptic protein class. In mammals, Crys are essential components of the circadian clock, but their role in other vertebrates is less clear. In invertebrates, Crys can function as circadian photoreceptors, or as components of the circadian clock, while in some species, both light-receptive and clock factor roles coexist. In the current study, we investigate the function of Cry proteins in zebrafish (*Danio rerio*), a freshwater teleost expressing 6 *cry* genes. Zebrafish peripheral circadian clocks are intrinsically light-sensitive, suggesting the involvement of Cry in light-resetting. Echinoderms (*Strongylocentrotus purpuratus*) represent the only class of deuterostomes that possess an orthologue (*SpuCry*) of the light-sensitive *Drosophila melanogaster* Cry, which is an important component of the light-resetting pathway, but also works as transcriptional repressor in peripheral clocks of fruit flies. We therefore investigated the potential of different zebrafish *cry* genes and *SpuCry* to replace the light-resetting and repressor functions of *Drosophila* Cry by expressing them in fruit flies lacking endogenous *cry* function. Using various behavioral and molecular approaches, we show that most Cry proteins analyzed are able to fulfill circadian repressor functions in flies, except for one of the zebrafish Crys, encoded by *cry4a*. Cry4a also shows a tendency to support light-dependent Cry functions, indicating that it might act in the light-input pathway of zebrafish.

Cryptochromes evolved from the ancient protein family of photolyases, which use light energy to repair ultraviolet-damaged DNA (Deppisch et al., 2022; Ozturk, 2017; Sancar, 2003). Most animal cryptochromes play a role in the circadian clock although their specific function varies dramatically. The *Drosophila-*type cryptochrome (type 1 Cry) functions as a circadian photoreceptor, at least within the pacemaker neurons of the central brain (Emery et al., 1998; Stanewsky et al., 1998). In contrast, the mammalian-type cryptochromes (type 2 Crys) have lost their ability to sense light, while they have retained their DNA-binding capacity and function as circadian repressors (Kume et al., 1999). Moreover, recent work suggests that both type 1 and type 2 Crys can sense the earth’s magnetic field (reviewed in the work of Merlin, 2023). Although the role of Cry as magneto sensor in flies has recently been questioned (Bassetto et al., 2023), there is considerable evidence for insect magnetosensitivity mediated by type 1 Crys using various paradigms by several independent groups (e.g., Bae et al., 2016; Fedele et al., 2014; Merlin, 2023; Wan et al., 2021; Yoshii et al., 2009). The proposed canonical mechanism suggests that light absorption by the Cry cofactor flavine adenine dinucleotide (FAD) initiates an electron-transfer cascade along a Cry tryptophane chain, resulting in formation of a radical pair, which is sensitive to magnetic fields (Hore and Mouritsen, 2016). As previously demonstrated for Cry activation by light, this could lead to conformational changes in the 52-amino-acid-long C-terminal tail of Cry, enabling interactions with other proteins, and thereby the possibility to signal the active state to other molecules and cells (Czarna et al., 2013; Fogle et al., 2015; Levy et al., 2013; Mazzotta et al., 2013; Peschel et al., 2009; Vaidya et al., 2013). Surprisingly, recent evidence indicates that the C-terminal tail alone (lacking FAD binding sites and the tryptophan chain) and even high concentrations of FAD alone can confer magnetic sensitivity to fly neurons, suggesting that Cry potentiates and transduces magnetic field signals, rather than sensing them (Bradlaugh et al., 2023).

Zebrafish (*Danio rerio*) contain circadian clocks throughout their bodies, and similar to peripheral clocks in *Drosophila melanogaster*, these clocks can be synchronized to light:dark cycles independently of each other *in vitro* (Frøland Steindal and Whitmore, 2019; Giebultowicz et al., 2000; Whitmore et al., 2000). Strikingly, even the circadian clock of an embryonic zebrafish cell line can be synchronized to light:dark cycles, indicating the existence of a cell autonomous photopigment (Dekens and Whitmore, 2008; Tamai et al., 2004). In *Drosophila*, peripheral circadian clock resetting is mediated by Cry, whereas the central brain clock is synchronized both by Cry and visual system photoreceptors (rhodopsins) (Ivanchenko et al., 2001; Stanewsky et al., 1998). The freshwater teleost *Danio rerio* encodes six different cryptochrome proteins. Based on sequence comparisons, Cry1a, Cry1b, Cry2a, and Cry2b are closely related to the mammalian type 2 Cry proteins (Deppisch et al., 2022; Oliveri et al., 2014). Zebrafish Cry3 and Cry4 each form a different group, suggesting the existence of a total of 3 Cry groups in teleosts (Kobayashi et al., 2000). Moreover, in contrast to zebrafish Cry1a, Cry1b, Cry2a, and Cry2b, zebrafish Cry3 and Cry4 expressed in human cell lines did not show repressor activity of Clock- and Bmal1-induced transcription, further suggesting that they form functionally distinct groups (Kobayashi et al., 2000; Liu et al., 2015). However, in zebrafish cells, Cry3 is expressed predominantly nuclear and shows potent repressor activity toward zebrafish *per1*- and *cry1a*-driven reporter gene transcription (Ferrer Prat, 2008). Maximum likelihood (ML) phylogenetic analysis also suggests a functional similarity between zebrafish Cry4 and *Drosophila* Cry (Kobayashi et al., 2000). In addition, zebrafish Cry4 is closely related to avian Cry4, which is both light and magneto-sensitive (Deppisch et al., 2022; Xu et al., 2021; Zoltowski et al., 2019). Moreover, zebrafish *cry1a* expression is induced by light and correlated to the magnitude of the phase shift of *per1* expression in a zebrafish cell line containing a functional circadian clock (Tamai et al., 2007). These results suggest that Cry1a and/or Cry4 may function as photoreceptors for circadian clock entrainment in zebrafish. In support of this, neither Cry1a nor Cry4 exhibit DNA repair activity, showing that they do not function as photolyases (Kobayashi et al., 2000). However, Cry1a, but not Cry4, represses CLK-BMAL-mediated transcription in reporter assays (Kobayashi et al., 2000; Tamai et al., 2007), suggesting that Cry1a may act in light detection, as a component of the light signal transduction pathway.

Interestingly, echinoderms (*Strongylocentrotus purpuratus*) represent the only class of deuterostomes that possess an orthologue of *Drosophila* Cry (Oliveri et al., 2014; Rubin et al., 2006; Yuan et al., 2007) supporting the idea that SpuCry may function as circadian photoreceptor in this phylum. However, both zebrafish and echinoderms also possess numerous opsin genes (e.g., up to 42 in zebrafish), some of which most likely play a role in light synchronization of circadian clocks (D’Aniello et al., 2015; Davies et al., 2015; Lesser et al., 2011; Raible et al., 2006), although functional evidence is lacking at this time. In order to test if *Danio rerio* and *S. purpuratus* candidate Crys possess photoreceptive functions, we tested if they can replace the function of *Drosophila* Cry. For this, we generated transgenic *Drosophila* expressing the heterologous *cry* genes in a genetic background lacking endogenous *cry* function (*cry^b^*) (Stanewsky et al., 1998). Using a similar approach, the function of human Cry1 and 4 types of crustacean Cry from *Daphnia magna* has been analyzed (Nitta et al., 2019; Vieira et al., 2012). Rather than performing a global analysis of zebrafish Cry proteins, the aim of this study was to explicitly test the potential photoreceptor roles for Cry1a and Cry4, as well as to address the debated role of Cry3 as a transcriptional repressor. While none of the zebrafish or sea urchin *cry* genes was able to restore normal light synchronization in *cry^b^* mutant flies, our results show that, with the exception of zebrafish *cry4*, all tested *cry* genes encode potent repressors of *period* transcription in *Drosophila*, indicating that they most likely function as circadian repressors, similar to mammalian type 2 Crys.

## Materials And Methods

### Cloning of *zCry* and *Spu-dCry* Into *Drosophila* Transformation Vectors

To generate *pUAST-attB-cry1a*, *cry3*, and *cry4*, the zebrafish *cry* genes were subcloned from *pGAD-cry1a*, *pGEM-cry3*, and *pBS-cry4* into *pUAST-attB* (Brand and Perrimon, 1993). The full coding sequence of the *SpuCry* gene (*S. purpuratus* genome 3.1: SPU_000282, WHL22.613873; *S. purpuratus* genome 5.0: LOC581225) was amplified using the Forw-EcoRI-CCGGAATTCATGCCTGGCGGTGCCT and Rev-XhoI-TCCGCTCGAGATTAAGAAAAAGGAACAAAC primers and a full-length cDNA clone derived from *S. purpuratus* total RNA at the stage of 33 hours after fertilization (early gastrula). A purified fragment was cloned into the *pGemT* vector (Promega) according to manufacturer’s instructions. Recombinant clones were sequenced using T7 and SP6 primers to confirm the correct fragment had been cloned. To generate *pUAST-attB-SpuCry*, *SpuCry* was subcloned from *pGemT-SpuCry* into *pUAST-attB*. All constructs were verified by sequencing before injection into fly embryos.

### Flies

Flies were raised in 12 h:12 h light-dark (LD) cycles on a standard *Drosophila* medium (0.7% agar, 1.0% soya flour, 8.0% polenta/maize, 1.8% yeast, 8.0% malt extract, 4.0% molasses, 0.8% propionic acid, 2.3% nipagen) at 25 °C and 40%-60% humidity. *Pdf-gal4* (Renn et al., 1999), *Clk856-gal4* (Gummadova et al., 2009), and *tim-gal4:27* (Kaneko and Hall, 2000) were crossed into a homozygous mutant *cry^b^* background (Stanewsky et al., 1998) using appropriate balancer chromosomes and dominant markers. *UAS-cry24.5* and *UAS-per:16* lines have been described (Blanchardon et al., 2001; Emery et al., 1998) and are located on chromosomes *2* and *3*, respectively. *pUAST-attB* vectors containing zebrafish *cry1a*, *cry3*, *cry4*, and *SpuCry* were transformed into *y^1^* *v^1^nos-Φ31*, *attP40/attP40* flies using standard procedures. Transformants of each *cry* gene were then crossed into a homozygous *cry^b^* mutant background. *BG-luc60* and *plo3b-1* transgenics are located on chromosome *1* and *3*, respectively, and have previously been described (Stanewsky et al., 1998, 2002).

### Behavioral Analysis

Analysis of locomotor activity of 4- to 5-day-old male flies was performed using the *Drosophila* Activity Monitor System (DAM; Trikinetics). Individual flies were placed into glass tubes filled with 2% agar and 4% sucrose and loaded into the DAM system. The monitors were located inside a light- and temperature-controlled incubator (Percival) where the fly’s activity was monitored for 1-2 weeks depending on different experimental conditions. Plotting of behavioral activity, rhythmicity, and period calculations was performed using a signal-processing tool-box (Levine et al., 2002) implemented in Matlab (MathWorks). For phase determination, activity data were transferred to an Excel macro (Microsoft), and the position (phase) of the evening activity peak for each individual fly was determined for every day of the experiment (phase plots in Figure 2A and 2B) as described (Sehadova et al., 2009). To calculate how long a certain genotype requires for re-synchronizing to the shifted LD cycle, daily activity profiles of individual flies were plotted, and the number of days where the evening peak showed transient delays before reaching a stable phase was determined manually for each fly (Figure 2C and 2D).

### Immunohistochemistry

Flies were entrained in 12 h:12 h LD at 25 °C for 3-4 days before fixation. Ten flies of each genotype were fixed at ZT21 or 2 h after a light pulse (LP) given at ZT19; therefore, all the flies were collected at the same time. After the 2.5-h fixation in 4% paraformaldehyde in phosphate buffered saline (PBS) + 0.1% Triton-X100, fly brains were dissected and washed in PBS + 0.1% Triton-X100, followed by incubation with primary antibodies as described (Chen et al., 2015). Rat anti-TIM (1:1000) (Rush et al., 2006), mouse anti-Pigment Disp﻿ersing Factor (PDF) (1:1000, Developmental Studies Hybridoma Bank, DSHB), rat anti-HA (1:1000, Roche), and secondary rat AlexaFluor-594 and mouse AlexaFluor-647 antibodies (1:400, Invitrogen) were applied. Mounted brains were scanned using a Leica TCS SP5 confocal microscope. Quantification of TIM signals was performed (Gentile et al., 2013) with minor modifications: Pixel intensity of stained neurons and background staining in each neuronal group was measured using Image J. Background signal was determined by taking the average signal of two surrounding fields of each neuronal group and was subtracted from the neuronal signal. For each group of clock neurons, at least 6 hemispheres from each genotype were measured. Data were normalized by setting the peak value to 1, and the value from each time point was then divided by the peak value.

### Bioluminescence Measurements

Luciferase expression of individual flies was measured as described (Stanewsky et al., 2002). Briefly, 2- to 3-day-old males were ether-anesthetized and loaded in a 96-well microtiter plate in which every other well contained 100 µl of 5% sucrose, 1% agar, and 15mM luciferin. Flies were measured in a Packard Topcount Multiplate Scintillation Counter for 6-7 days during 12 h:12 h LD and DD at 25 °C as indicated in the figures. Data were plotted using BRASS software (Version 2.1.3) (Locke et al., 2005) and analyzed using Chronostar software (Klemz et al., 2017). In particular, data were first de-trended using a running average with a 24-h window. After trendline subtraction, data were subjected to a sinus fit operation, and the resulting curves were plotted in Figure 4B (see Klemz et al., 2017 for details).

## Results

In order to determine the photoreceptive potential of the different Cry proteins, we stably introduced them into the *Drosophila* germline using Φ31 C-mediated integration (see Materials and Methods). This technology allows integration into an identical position in the genome, thereby precluding differences in expression levels due to position effects associated with a particular chromosomal site. To facilitate transgene combination with a mutation of the *Drosophila cry* gene located on chromosome *3*, we chose the *attP40* landing site situated on chromosome *2* (Markstein et al., 2008). After successful transformation, 4 transgenic zebrafish and sea urchin *cry* lines (*cry1a*, *cry3*, *cry4*, *SpuCry*) were crossed into the *cry^b^* mutant background (Stanewsky et al., 1998). The transgenes contain *UAS* sequences allowing transcriptional activation of each *cry* gene by introducing the yeast GAL4 transcription factor (Brand and Perrimon, 1993). It has previously been shown that expression of *dcry* in all *timeless* (*tim*) expressing clock cells robustly rescues *cry^b^* molecular and behavioral phenotypes (Emery et al., 2000b), and we therefore expressed the zebrafish and sea urchin *cry* genes using the same *tim-gal4* line. In addition, we used the *Clk856-gal4* driver, which, like *tim-gal4*, is expressed in all clock neurons but lacks expression in peripheral clocks and glia cells (Gummadova et al., 2009). Finally, in some of the assays, we applied an even more restricted driver (*Pdf-gal4*), which is only expressed in 16 of the overall ~150 clock neurons in the fly brain (Renn et al., 1999). We then asked if the individual heterologously expressed *cry* transgenes were able to rescue any of the phenotypes caused by *cry^b^* . As a positive control, we also expressed *Drosophila cry* in a *cry^b^* mutant background using the same set of clock cell *gal4* drivers.

### Heterologous Zebrafish and Sea Urchin *cry* Expression in *cry^b^* Mutants Does Not Restore Circadian Clock Sensitivity to Constant Light

**Figure 1.**
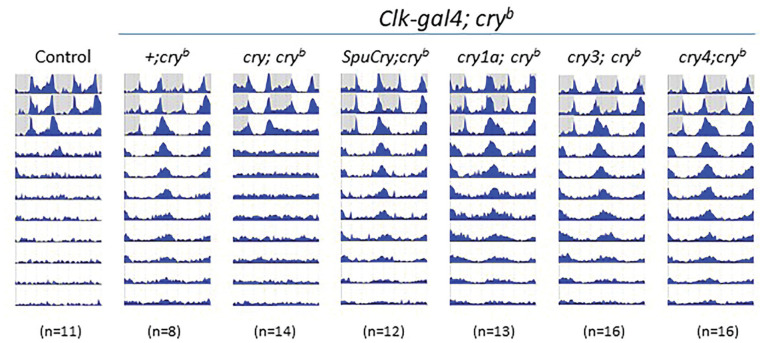
Zebrafish and sea urchin cryptochromes do not abolish constant-light rhythmicity induced by *cry^b^*. Male flies were exposed to 2 days of 12 h:12 h LD before being released into LL (~1500 lux 25 °C). Double-plotted actograms show average activity of the genotypes indicated above the plots (progeny of *Clk-gal4; *cry^b^** flies crossed to *UAS-cry; *cry^b^** or +; *cry^b^* flies). *cry* ^+^ control flies are *y w*. White areas indicate “lights-on,” and gray areas, “lights-off.” Note that wild-type (*y w*) and *Clk-gal4; UAS-cry; *cry^b^** flies become arrhythmic in LL, while *cry^b^* flies, as well as those expressing zebrafish or sea urchin *cry* genes, stay rhythmic. Similar results were obtained with the more restricted *Pdf-gal4* driver and at lower light intensities (see Supplementary Table S1).

The circadian clock of *Drosophila* fails to operate in constant light (LL), presumably because of constitutive light-dependent degradation of one of its key components, the clock protein Timeless (Tim) (Price et al., 1995; Zeng et al., 1996). As a consequence, while *Drosophila* locomotor activity rhythms are sustained in conditions of constant darkness (DD), wild-type flies become arrhythmic in LL and constant temperature (Konopka et al., 1989; Konopka and Benzer, 1971). Interfering with light-input pathways to the clock can restore clock function in LL, leading to molecular and behavioral rhythmicity (Chen et al., 2011; Emery et al., 2000a), while LL rhythmicity induced by the *cry^b^* mutation can be reversed to wild-type LL arrhythmicity by driving *UAS-cry* expression in all clock cells (Emery et al., 2000b). As a quick and straightforward assay to test if the various *cry* genes can replace light-dependent Cry functions in flies, we exposed *cry^b^* mutant flies heterologously expressing one of the different *cry* genes in all clock neurons (*Clk856-gal4/UAS-cry; cry^b^/ *cry^b^**) to bright LL (~1500 lux). As expected, wild-type flies were arrhythmic in LL, while homozygous *cry^b^/ *cry^b^** flies displayed robust rhythmicity (Figure 1, Supplementary Table S1). The LL-rhythmicity of *cry^b^* mutants could be fully rescued by driving *Drosophila UAS-cry* expression with *Clk856-gal4*. Based on sequence homology to *Drosophila Cry*, we predicted that *SpuCry* and zebrafish *Cry4* could at least partially replace its function, but *cry^b^* flies expressing these *cry* genes in all clock neurons remained thoroughly rhythmic in LL. As expected, none of the more distantly *Drosophila-*related *cry* genes (zebrafish *cry1a* and *cry3*) restored LL arrhythmicity (Figure 1). Similar results were obtained in dim LL (~100 lux) and with the *Pdf-gal4* driver, where expression of *Drosophila cry* resulted in 50% of LL arrhythmic flies as previously reported (Emery et al., 2000b) and none of the other *cry* genes had any effect (Supplementary Table S1). To rule out whether the LL-assay may not be suitable to detect potential partial photoreceptive functions of the heterologously expressed *cry* genes, we next turned to a more sensitive assay.

### Heterologous Zebrafish and Sea Urchin Cry Expression Does Not Rescue Slow Resynchronization of *cry^b^* Mutants to Altered LD Cycles

**Figure 2.**
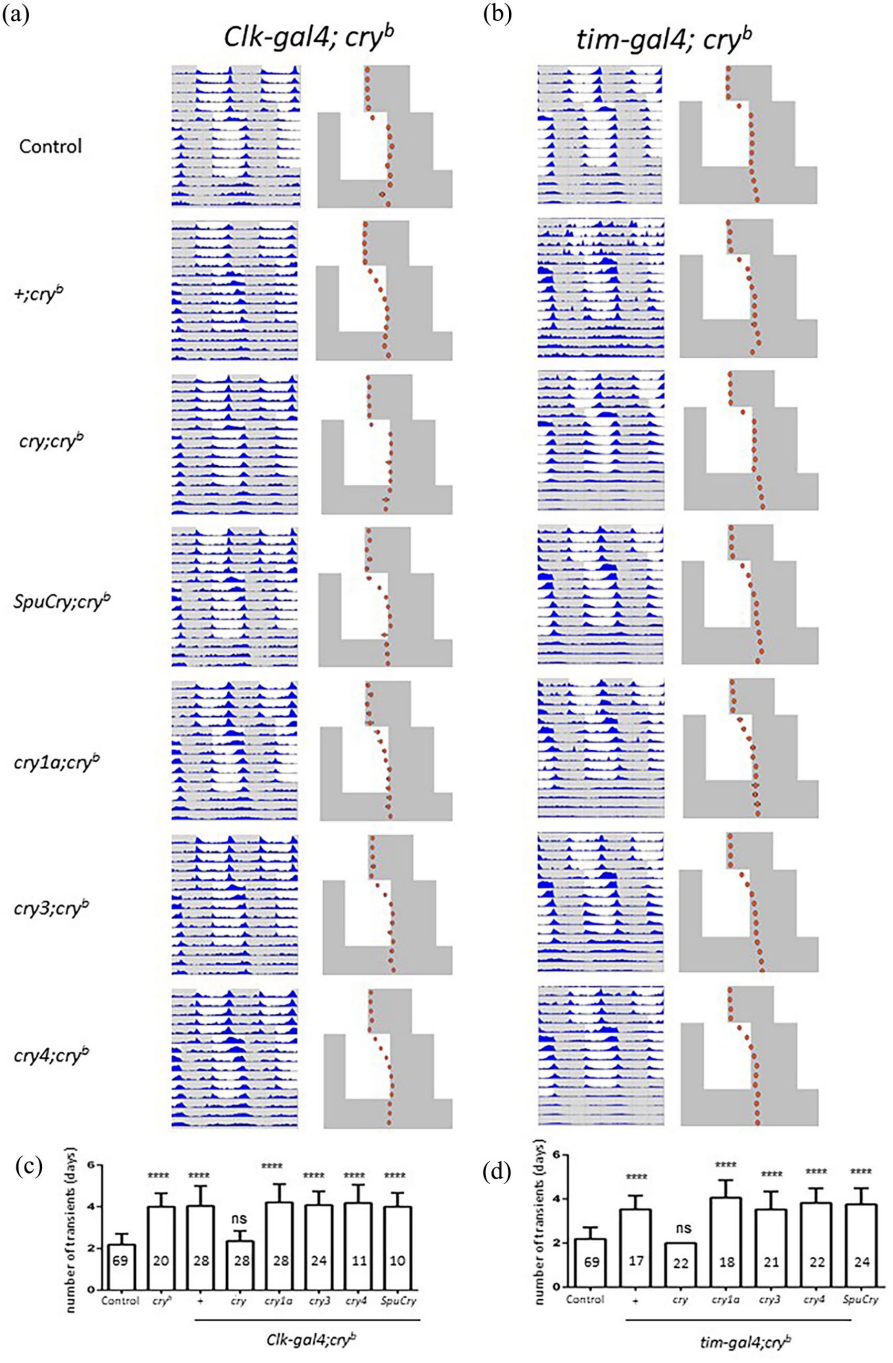
Zebrafish and sea urchin Cryptochromes do not enhance slow resynchronization of *cry^b^* mutants to LD cycles. (a-d) Male flies were exposed to 5 (a) or 4 (b) days of 12 h:12 h LD before delaying the LD cycle by 6 h. After 7 days in this delayed LD cycles, flies were released into DD for 3-4 days. (a, b) Double-plotted actograms on the left show average activity during the entire experiment. Phase plots on the right indicate the daily position of evening activity peak, with error bars indicating SEM. White portions indicate “lights-on,” and gray areas, “lights-off.” “Control” flies are *y w* combined with the progeny of *Clk-gal4; *cry^b^** (a) or *tim-gal4; *cry^b^** (b) flies crossed to *y w*. “+; *cry^b^* ” flies are progeny of *Clk-gal4; *cry^b^** (a) or *tim-gal4; *cry^b^** (b) flies crossed to *cry^b^*. All other genotypes contain one copy of *Clk-gal4; *cry^b^** (a) or *tim-gal4; *cry^b^** (b) as indicated on the top, plus one copy of a *UAS-cry* transgene (as indicated on the left) in a homozygous *cry^b^* mutant background. (c, d) Quantification of the days required for re-entrainment for each of the genotypes shown in (a) and (b). *cry^b^* controls were *Clk-gal4; *cry^b^** (c) or *tim-gal4; *cry^b^** (d) flies crossed to *cry^b^* (+), and flies from a homozygous mutant *cry^b^* stock (*cry^b^*) (c). Numbers within bars indicate *n.* Error bars indicate SEM. Significant differences between all genotypes and the controls were determined using the non-parametric Tukey test followed by Dunnett’s test (****p < 0.0001, ns: not significant).

Resynchronization to altered LD cycles (i.e. a jetlag assay) is a very sensitive behavioral assay to determine functionality of the different light-input pathways to the circadian clock. For example, compared to wild-type flies, which require only 1-2 days to resynchronize their behavioral activity pattern to an 8-h-delayed LD cycle, *cry^b^* mutants need 4-5 days, while flies with an additionally impaired visual system (*norpA^P41^* *cry^b^*) require >7 days to achieve this task (Emery et al., 2000b). Because it allows for detection of partially functional light input to the circadian clock, we exposed *cry^b^* mutant flies expressing one of the different *cry* genes in all clock cells to such a jetlag assay. In particular, *Clk856-gal4/UAS-cry; *cry^b^*/*cry^b^** flies were first kept in a 12 h:12 h LD cycle for 5 days, after which the LD cycle was delayed by 6 h. After exposure to this delayed LD cycle for 7 days, flies were released into DD for an additional 3-5 days. During the first 5 days, flies from all genotypes synchronized their activity pattern to the LD cycle, with activity peaks in the morning and evening. In addition, all flies “anticipated” the environmental light transitions in the morning and evening by increasing their locomotor activity several hours before the actual light transition, indicative of light synchronization of the underlying circadian clock (Wheeler et al., 1993). As expected, control flies rapidly adjusted their activity pattern to the 6-h-delayed LD regime within 1-2 days, while homozygous *cry^b^/ *cry^b^** flies required ~4-5 days before adjusting their evening activity peak to the shifted LD regime (Figure 2a). The slow resynchronization of *cry^b^* mutants could be fully rescued by driving *Drosophila UAS-cry* expression with *Clk856-gal4* (Figure 2a). We predicted that in this more sensitive assay, *SpuCry* and zebrafish *cry4* would at least partially restore *cry* function in the fly and speed up resynchronization to delayed LD cycles in *cry*^b^ mutants, but this was not the case (Figure 2a). Quantifying the days required to reach a stable activity pattern in the shifted LD regime (i.e., after the jetlag), revealed no significant differences between homozygous *cry*^b^*/cry*^b^ flies expressing none and those expressing any of the different *cry* genes (Figure 2c). To rule out the possibility that *cry* expression driven by *Clk856-gal4* may not be strong enough, or spatially too restricted, we repeated these experiments using *tim-gal4*. Again, no improvement of light resynchronization was induced by any of the heterologous *cry* genes, while expression of *Drosophila cry* resulted in wild-type behavior (Figure 2b and 2d). Taken together, our results suggest that the zebrafish and sea urchin *cry* genes analyzed here are not able to restore light- and *cry-*dependent behavior in flies (Figures 1 and 2).

### Heterologous Zebrafish and Sea Urchin Cry Expression Does Not Restore Light-Dependent Timeless Degradation in Clock Neurons of *cry*^b^ Mutants

**Figure 3.**
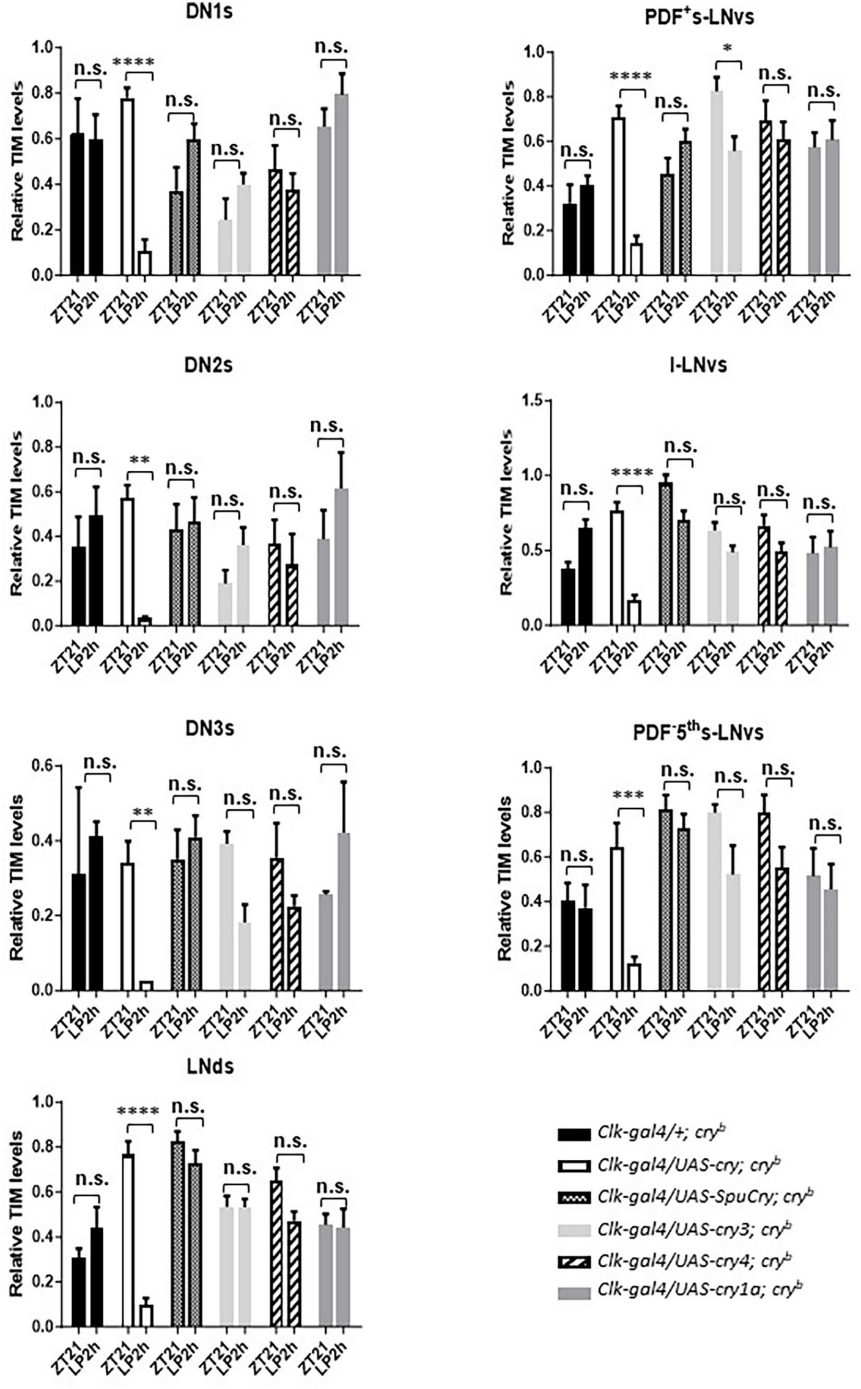
Zebrafish and sea urchin cryptochromes do not restore robust light-dependent Tim degradation in *cry^b^* mutants. Male flies were kept in 12 h:12 h LD cycles, before being exposed to a 2-h light pulse (LP, ~1500 lux) starting at ZT19. Brains of light-pulsed and non-pulsed control flies were dissected at ZT21 and incubated with Tim antibodies. Tim levels in all clock neuronal groups (apart from the LPN) were determined and compared between light-pulsed and dark controls in the genotypes indicated. Note that in *cry^b^* mutants expressing *Drosophila cry* in all clock neurons (*Clk-gal4/UAS-cry; *cry^b^**), Tim levels are drastically reduced after the LP compared to dark controls. In contrast, Tim levels are always high in *cry^b^* mutants expressing no or any of the zebrafish or sea urchin *cry* genes. Only zebrafish *cry4*-expressing flies show a consistent (yet not significant) reduction of Tim in all clock neuronal groups after the LP. At least 12 brain hemispheres were analyzed for each condition and genotype. To test statistical significance of intensity differences between the two time points, a two-way ANOVA with Sidak’s post-comparison was performed. *p < 0.05, **p < 0.01, ***p < 0.001, ****p < 0.0001, and n.s., no significance. Error bars indicate SEM.

To investigate if any of the Cry proteins encoded by the sea urchin and zebrafish *cry* genes can support light responses of the molecular clock, we measured light-induced degradation of Tim protein in clock neurons. The various *cry* genes were expressed in *cry^b^* mutant flies using the *Clk856-gal4* driver, and Tim levels were determined by immunofluorescence late at night, when Tim levels reach their maximum (at Zeitgeber Time [ZT] 21, meaning 3 h before the lights came on in a 12 h:12 h LD cycle). These values were then compared to Tim levels in flies which were exposed to a 2 h of bright LP starting at ZT19. As expected, in *cry^b^* mutant flies, Tim levels in all clock neurons were similar between the control and LP-treated flies (Yoshii et al., 2015). In contrast, *Clk856-gal4*-driven expression of *Drosophila cry* led to a strong reduction of Tim in all clock neuronal groups, indicating a rescue of Tim stabilization induced by *cry^b^* (Figure 3). In agreement with our behavioral results (Figures 1 and 2), none of the zebrafish or sea urchin Cry proteins induced a clear reduction of Tim levels in the clock neurons of LP-treated flies. Although only the expression of zebrafish *cry4* consistently showed a trend toward reduced Tim levels in all neuronal groups in the LP-treated flies, the difference to the non-pulsed controls was not significant, indicating that none of the heterologously expressed Cry proteins supports light-dependent Tim degradation.

### Heterologous Zebrafish and Sea Urchin Cry Expression Does Not Restore Light-Dependent *Period-Luciferase* Oscillations in Peripheral Clock Cells of *cry*^b^ Mutants

**Figure 4.**
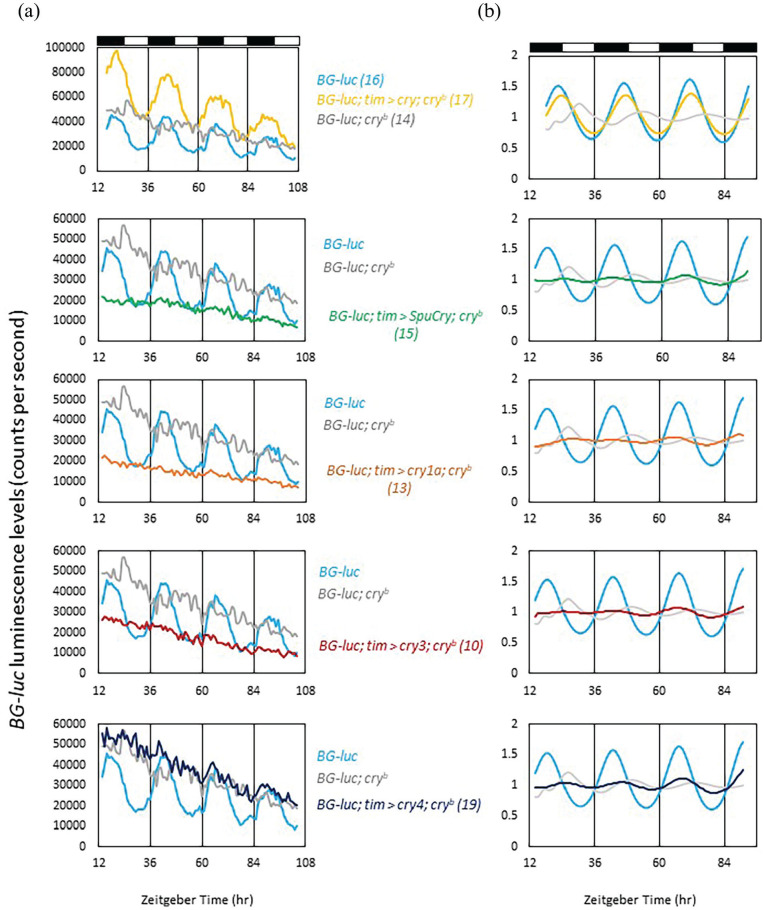
Zebrafish and sea urchin Cryptochromes do not restore light-dependent *period-luciferase* oscillations in *cry^b^* mutants. Male *BG-luc* flies were placed individually in the wells of microtiter plates filled with food and luciferin. Bioluminescence was measured hourly using a TopCount reader (PerkinElmer) during 3 days of LD (a, b), followed by 3 days of DD (a) as described in the Materials and Methods section. Except for *BG-luc* control flies (yellow), all other genotypes were homozygous mutant for *cry^b^*, which diminishes the robust bioluminescence oscillations emitted by *BG-luc* flies during LD (compare yellow and gray tracks in the upper panels of (a) and (b) (Stanewsky et al., 1998). To test the ability to restore *BG-luc* oscillations, *Drosophila cry* and the 4 heterologous *cry* genes were expressed in the *BG-luc; *cry^b^** mutant background using *tim-gal4*. (a) Raw bioluminescence data showing that except for *Drosophila cry* none of the tested *cry* genes is able to restore robust *BG-luc* oscillations. (b) Data of the LD part only were de-trended and cosine-fitted (see Material and Methods) to reveal more subtle differences. Dark and white bars above the plots indicate dark and light periods, respectively. Numbers in parentheses indicate *n*.

*Drosophila Cry* also mediates molecular synchronization of peripheral clock cells to LD cycles (Ivanchenko et al., 2001). In fact, the original *cry^b^* mutation was isolated in a screen for altered *period-luciferase* (*per-luc*) oscillations in peripheral clock cells during LD cycles. While wild-type flies displayed robust *per-luc* oscillations in LD, *cry^b^* abolished these oscillations (Stanewsky et al., 1998). Because daily temperature cycles restored Per and Tim protein as well as *per-luc* oscillations in *cry^b^* mutant flies, it followed that Cry is required for light-resetting of peripheral circadian clocks in flies (Glaser and Stanewsky, 2005; Ivanchenko et al., 2001; Stanewsky et al., 1998). To test if the zebrafish and sea urchin Crys support light synchronization of peripheral clocks in flies, we expressed them individually in *cry^b^* mutant flies expressing the same *per-luc* reporter (*BG-luc*, containing 4 kb of 5’-flanking regulatory sequences and about two-third of the PER coding region fused to *luciferase* cDNA) used to isolate *cry^b^*. Flies were placed individually in the wells of 96-well microtiter plates, and luminescence originating from each fly was measured once per hour during 2.5 days of LD followed by 4 days of DD. As expected, *cry^b^* mutants showed no or low-amplitude *per-luc* oscillations when looking at average raw bioluminescence counts or de-trended and curve-fitted data, respectively, while wild-type flies expressed robust and light-dependent luciferase rhythms (Figure 4a and 4b). Expressing *Drosophila Cry* with the *tim-gal4* reporter restored *per-luc* rhythms in *cry^b^* mutant flies, confirming that this assay can be used to test the function of the zebrafish and sea urchin Crys in light synchronization of peripheral clocks (Figure 4A and 4B). As described for clock neuronal light responses earlier, only zebrafish *cry4* showed limited ability to restore *per-luc* rhythmicity in *cry^b^* flies (Figure 4B). Interestingly, *SpuCry*, zebrafish *cry1a*, and *cry3* led to trough levels of *per-luc* expression during the LD part of the experiment, suggesting that the respective proteins can act as repressors of *per* expression (see below).

### Zebrafish Cry1a, Cry3, and SpuCry Can Function as Transcriptional Repressors of *Period* Expression in *Drosophila*

**Figure 5.**
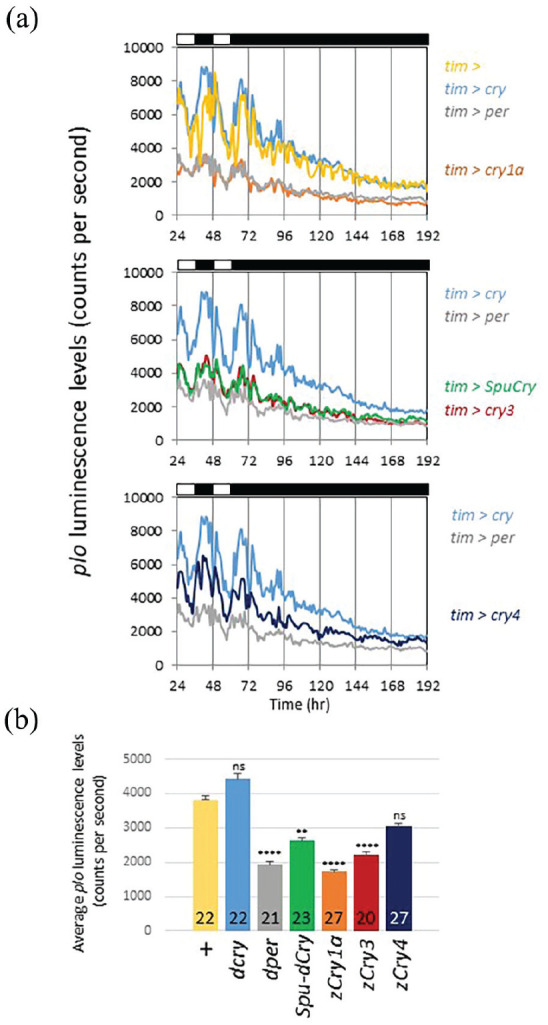
Zebrafish Cry1a, Cry3, and SpuCry function as transcriptional repressors of *period-luciferase* expression in *Drosophila*. The ability to repress *per* transcription was tested by overexpressing the various *cry* genes in flies carrying the transcriptional *per-luc* reporter *plo*, which contains only 5’-flanking regulatory DNA sequences of the *per* gene (Brandes et al., 1996). Bioluminescence emanating from male *plo* flies was measured during 2 days of LD followed by 5 days of DD as described in the legend of Figure 4. Control flies (yellow “*tim* *>*” in [a] and “+” in [b]) carry 1 copy of *tim-gal4* on chromosome *2* and 1 copy of *plo* on chromosome *3*. Test flies in addition carry 1 copy of the respective *UAS-cry* construct on chromosome *2*. As a positive control for repression, we also crossed *UAS-per* (on chromosome *3*) to *tim-gal4; plo* flies (gray in each panel). (a) Raw averaged bioluminescence recordings from flies with the genotypes indicated to the right. Upper panel: Controls (yellow and gray) and *tim-gal4; plo* flies expressing *Drosophila cry* (blue), and zebrafish *cry1a* (orange), a strong repressor. Middle panel: *tim-gal4; plo* flies expressing *SpuCry* (green) and zebrafish *cry3* (red) showing medium repression. Lower panel: *tim-gal4; plo* flies expressing zebrafish *cry4*, encoding a weak repressor. White and black bars above each panel indicate times of light and darkness. (b) Quantification of the average expression level for each genotype for the data is shown in (a). Genotypes and color codes as in (a). Numbers in bars indicate *n*, and error bars SEM. Data represent results from 3 independent experiments. Significant differences between all genotypes and the *tim-gal4; plo* controls (“+”) were determined using the non-parametric Tukey test followed by Dunnett’s test (**p < 0.005, ****p < 0.0001, ns: not significant).

The mammalian type 2 Cry proteins function as essential, light-independent repressor proteins in the circadian clock. Mouse Cry1 and Cry2 repress transcription by binding to the transcription factors Clock and Bmal1 (Shearman et al., 2000). To directly test the possibility that the zebrafish and sea urchin Cry proteins can act as repressors of *per* transcription, we applied a *per-luc* reporter (*plo*), which faithfully reports *per* transcriptional rhythms (Brandes et al., 1996; Stanewsky et al., 1997). This *plo* reporter contains the same 4-kb upstream regulatory DNA sequences as *BG-luc*, which are directly fused to the *luciferase* gene (so no per coding sequences) (Brandes et al., 1996). *plo* transgenics exhibit robust oscillations in luminescence in LD, which rapidly dampen in DD (Figure 5). We expressed the various *cry* genes in *plo* flies using the *tim-gal4* driver to see if this would cause a reduction of overall *plo* luminescence levels. As a positive control, we also expressed *Drosophila UAS-per* using the same *tim-gal4* driver because Per is a known repressor of its own transcription (Zeng et al., 1994). As expected, and in agreement with previous observations (Zeng et al., 1994), overexpression of Per resulted in a drastic reduction of *plo* luminescence levels in the LD and DD parts of the experiment, while rhythmic expression was only maintained during LD (Figure 5a and 5b). Although *Drosophila Cry* has been shown to act as a transcriptional repressor (Collins et al., 2006), we did not observe a reduction of *plo* oscillation amplitude, nor decreased levels after overexpressing *Drosophila cry*, indicating that *Drosophila Cry* does not act as a repressor of *per* expression in the *tim-*expressing cells contributing to the bioluminescence signal. Strikingly, expression of zebrafish *cry1a*, *cry3*, and *SpuCry* had essentially the same effect on *plo* expression as overexpression of Per, strongly indicating that the Cry proteins encoded by these three genes can function as repressor of *per* transcription in *Drosophila*. The result for *cry3* was surprising given its lack of repressive function when expressed in human cells (Kobayashi et al., 2000; Liu et al., 2015). Compared to wild-type controls, zebrafish *cry4* also reduced *plo* levels, but this reduction was not significant, indicating that Cry4 has only weak repressor function in flies, if any (Figure 5a and 5b). In agreement with this result, Cry4 mainly localizes to the cytoplasm when expressed in fly clock neurons (Supplementary Figure S1).

## Discussion

Our results confirm that zebrafish Cry1a and Cry3, which are more closely related to mammalian type 2 Crys (Oliveri et al., 2014), indeed can function as transcriptional repressors in flies *in vivo*. Contrary to our expectations, SpuCry is not able to restore light-dependent Cry functions in *Drosophila* but is able to function as a potent repressor of *per* transcription. The situation with regard to zebrafish Cry4 is more complicated: Phylogenetic DNA sequence analysis suggested that out of the six zebrafish *Cry* genes, the protein encoded by *cry4* is the one most closely related to *Drosophila Cry* (Oliveri et al., 2014), suggesting that it may have photoreceptive function. Although our behavioral studies do not support this idea (Figures 1 and 2), our molecular analyses raise the possibility that zebrafish Cry4 indeed has photoreceptive functions. First, *cry4* expression showed some ability to restore *per-luc* rhythmicity in *cry^b^* flies (Figure 4B), in contrast to other cryptochromes (except for *Drosophila Cry*). Second, when expressed in peripheral clock cells, only *Drosophila Cry* and zebrafish Cry4 did not show significant transcriptional repressor activity. Third, only zebrafish Cry4 showed a trend toward light-dependent TIM reduction across all clock neuronal cell types analyzed, although this effect was not significant. One possibility for the strongly reduced (zebrafish Cry4) or absent (SpuCry) light-dependent Cry functions in the *Drosophila* host could be reduced stability of the heterologously expressed proteins. We do not think that this is the case though because we did see clear repressive effects of SpuCry on *per* transcription (Figure 5). For zebrafish Cry4, which did not show significant effects on *per* transcription, we observed modest effects on light-dependent Tim stability and *per-luc* cycling, indicating that the Cry4 protein is also stable in flies. Moreover, we directly demonstrate that Cry4 is detectable when expressed in clock neurons (Supplementary Figure S1). Overall, expression analysis for zebrafish Cry4 (Supplementary Figure S1) and the repressor function observed for the other heterologous Cry proteins (Figure 4) indicate that they are stably expressed in fly clock cells. This makes it unlikely that the lack of restoring light-dependent Cry functions is due to insufficient stability of the zebrafish and sea urchin Cry proteins in fly tissues. With regard to subcellular localization, available data for *Drosophila Cry* indicate nuclear and cytoplasmic localization, with more prominent accumulation in the latter compartment (Yoshii et al., 2008). Since *Drosophila Cry* mediates light-dependent Tim degradation in the morning when Tim is nuclear (Shafer et al., 2002), it seems clear that nuclear Cry is responsible for this degradation. Nuclear localization and repressor activity for Cry3 in zebrafish cells have been shown before (Ferrer Prat, 2008), and here we show nuclear and cytoplasmic expression of zebrafish Cry4 (Supplementary Figure S1) as well as repressor activity for all other Crys (Figure 3). This suggests that all Cry proteins analyzed in the current study are located in the nucleus, and therefore principally in the right place to mediate light-dependent Tim degradation. However, direct expression analysis would be required to ultimately determine the stability and subcellular localization dynamics of all Cry proteins investigated in this study.

Our results, along with previous studies, strongly support the idea that genome duplication in fish, which has led to the increase in *cry* genes, has allowed cryptochromes to play a variety of different roles within the clock mechanism. Zebrafish Cry4 has the potential to play a photoreceptive role in the fish system, in conjunction with the large number of non–image-forming photoreceptors expressed in fish. Zebrafish *cry1a* expression is robustly light-induced, and Cry1a clearly binds to CLOCK and BMAL proteins to stop their active dimerization (Tamai et al., 2007). As such, Cry1a acts as a key component of the light signal transduction cascade. Zebrafish Cry3 on the other hand has a clear transcriptional repressive function within the clock mechanism, and as such is likely to be a core clock component.

Considering that the various Cry proteins are expressed heterologously in the fly, it is likely that their potential binding partners are too diverse compared to those present in zebrafish or sea urchin, to reveal their true endogenous function. In other words, a subtle light-dependent function of zebrafish Cry4 in the fly may indicate a more prominent photoreceptive function in zebrafish. Ultimately, intraspecies *in vivo* studies will be necessary to fully resolve the function of the various Cry proteins.

## Supplemental Material

sj-pptx-1-jbr-10.1177_07487304241228617 – Supplemental material for Functional Analyses of Four Cryptochromes From Aquatic Organisms After Heterologous Expression in Drosophila melanogaster Circadian Clock Cells Supplemental material, sj-pptx-1-jbr-10.1177_07487304241228617 for Functional Analyses of Four Cryptochromes From Aquatic Organisms After Heterologous Expression in Drosophila melanogaster Circadian Clock Cells by Chenghao Chen, T. Katherine Tamai, Min Xu, Libero Petrone, Paola Oliveri, David Whitmore and Ralf Stanewsky in Journal of Biological Rhythms
