# Supplementary material for: Functional Analyses of Four Cryptochromes From Aquatic Organisms After Heterologous Expression in Drosophila melanogaster Circadian Clock Cells
Source: J Biol Rhythms. 2024 Mar 28;39(4):365–78. doi: 10.1177/07487304241228617 (PMC11292970; doi:10.1177/07487304241228617)
Supplement: sj-pptx-1-jbr-10.1177_07487304241228617 – Supplemental material for Functional Analyses of Four Cryptochromes From Aquatic Organisms After Heterologous Expression in Drosophila melanogaster Circadian Clock Cells [file sj-pptx-1-jbr-10.1177_07487304241228617.pptx]

## Slide 1
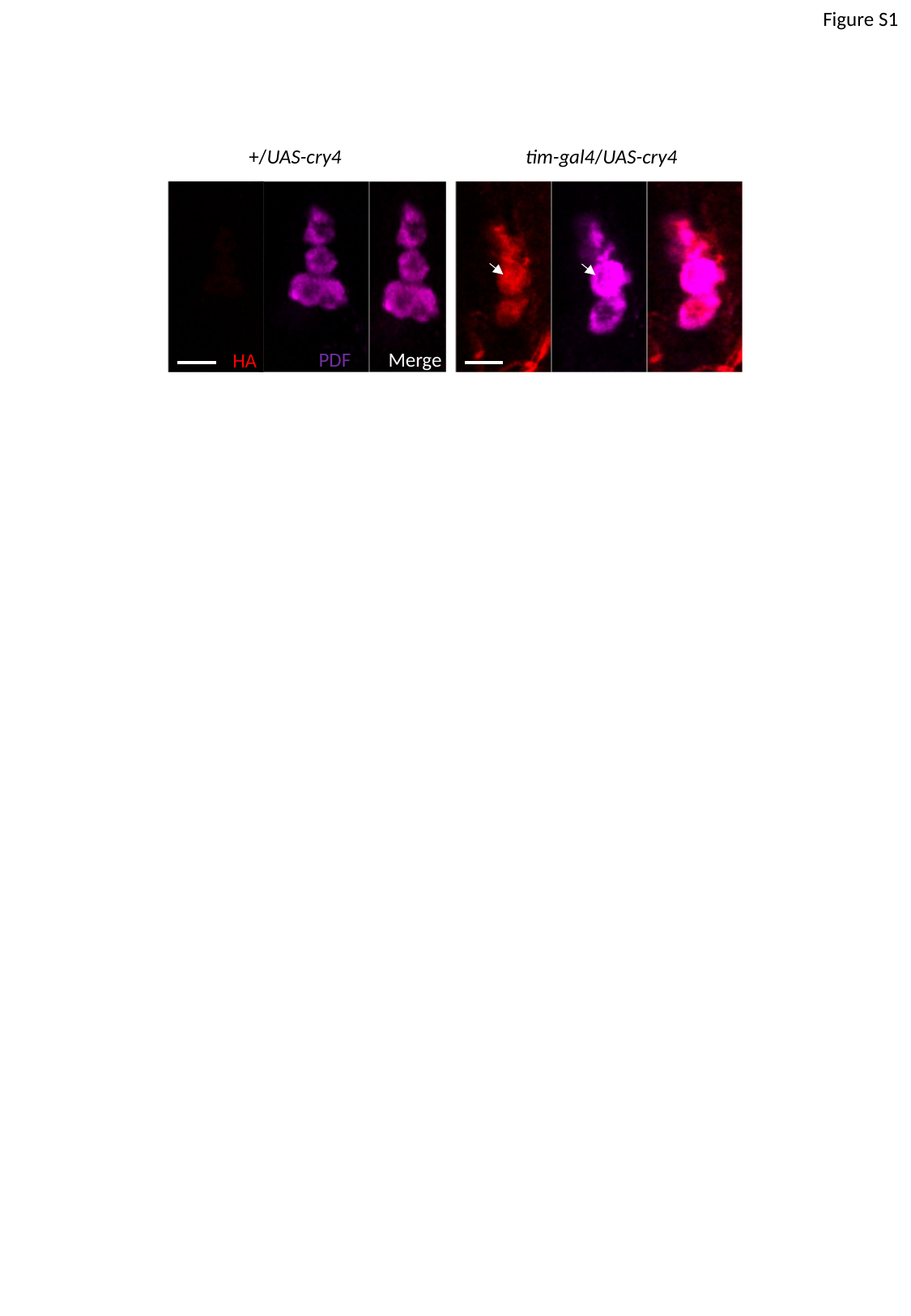

Figure S1
+/UAS-cry4
tim-gal4/UAS-cry4
Merge
PDF
HA

## Slide 2
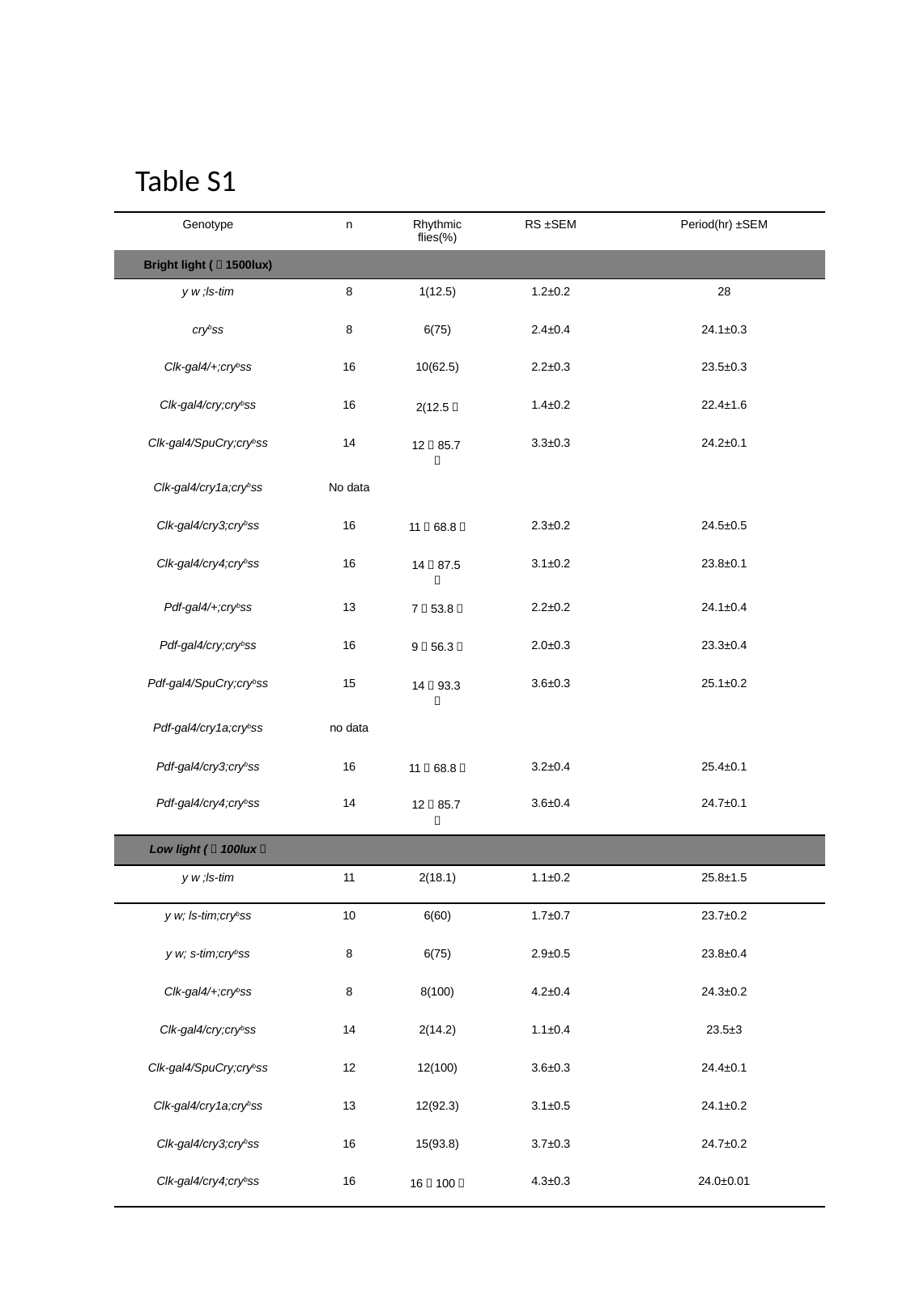

Table S1
| Genotype | n | Rhythmic flies(%) | RS ±SEM | Period(hr) ±SEM |
| --- | --- | --- | --- | --- |
| Bright light (～1500lux) | | | | |
| y w ;ls-tim | 8 | 1(12.5) | 1.2±0.2 | 28 |
| crybss | 8 | 6(75) | 2.4±0.4 | 24.1±0.3 |
| Clk-gal4/+;crybss | 16 | 10(62.5) | 2.2±0.3 | 23.5±0.3 |
| Clk-gal4/cry;crybss | 16 | 2(12.5） | 1.4±0.2 | 22.4±1.6 |
| Clk-gal4/SpuCry;crybss | 14 | 12（85.7） | 3.3±0.3 | 24.2±0.1 |
| Clk-gal4/cry1a;crybss | No data | | | |
| Clk-gal4/cry3;crybss | 16 | 11（68.8） | 2.3±0.2 | 24.5±0.5 |
| Clk-gal4/cry4;crybss | 16 | 14（87.5） | 3.1±0.2 | 23.8±0.1 |
| Pdf-gal4/+;crybss | 13 | 7（53.8） | 2.2±0.2 | 24.1±0.4 |
| Pdf-gal4/cry;crybss | 16 | 9（56.3） | 2.0±0.3 | 23.3±0.4 |
| Pdf-gal4/SpuCry;crybss | 15 | 14（93.3） | 3.6±0.3 | 25.1±0.2 |
| Pdf-gal4/cry1a;crybss | no data | | | |
| Pdf-gal4/cry3;crybss | 16 | 11（68.8） | 3.2±0.4 | 25.4±0.1 |
| Pdf-gal4/cry4;crybss | 14 | 12（85.7） | 3.6±0.4 | 24.7±0.1 |
| Low light (～100lux） | | | | |
| y w ;ls-tim | 11 | 2(18.1) | 1.1±0.2 | 25.8±1.5 |
| y w; ls-tim;crybss | 10 | 6(60) | 1.7±0.7 | 23.7±0.2 |
| y w; s-tim;crybss | 8 | 6(75) | 2.9±0.5 | 23.8±0.4 |
| Clk-gal4/+;crybss | 8 | 8(100) | 4.2±0.4 | 24.3±0.2 |
| Clk-gal4/cry;crybss | 14 | 2(14.2) | 1.1±0.4 | 23.5±3 |
| Clk-gal4/SpuCry;crybss | 12 | 12(100) | 3.6±0.3 | 24.4±0.1 |
| Clk-gal4/cry1a;crybss | 13 | 12(92.3) | 3.1±0.5 | 24.1±0.2 |
| Clk-gal4/cry3;crybss | 16 | 15(93.8) | 3.7±0.3 | 24.7±0.2 |
| Clk-gal4/cry4;crybss | 16 | 16（100） | 4.3±0.3 | 24.0±0.01 |
